# Autoantibodies targeting interferons and GM-CSF are associated with adverse outcome risk, comorbidities, and pathogen in community-acquired pneumonia

**DOI:** 10.3389/fimmu.2024.1459616

**Published:** 2024-11-13

**Authors:** Jakob Hjorth Von Stemann, Arnold Matovu Dungu, Maria Vispe Laguarda, Camilla Koch Ryrsø, Maria Hein Hegelund, Daniel Faurholt-Jepsen, Rikke Krogh-Madsen, Morten Bagge Hansen, Birgitte Lindegaard, Sisse Rye Ostrowski

**Affiliations:** ^1^ Department of Clinical Immunology, Copenhagen University Hospital, Rigshospitalet, Copenhagen, Denmark; ^2^ Department of Pulmonary and Infectious Diseases, Copenhagen University Hospital, North Zealand, Denmark; ^3^ Centre for Physical Activity Research, Copenhagen University Hospital, Rigshospitalet, University of Copenhagen, Copenhagen, Denmark; ^4^ Department of Infectious Diseases, Copenhagen University Hospital, Rigshospitalet, Copenhagen, Denmark; ^5^ Department of Clinical Medicine, Copenhagen University Hospital, Rigshospitalet, Copenhagen, Denmark; ^6^ Department of Infectious Diseases, Copenhagen University Hospital, Hvidovre, Denmark; ^7^ Department of Clinical Medicine, Faculty of Health and Medical Sciences, University of Copenhagen, Copenhagen, Denmark

**Keywords:** community-acquired pneumonia (CAP), coronavirus disease 2019, cytokine autoantibody, type 1 interferon, interleukin-10, granulocyte-macrophage colony stimulating factor

## Abstract

**Introduction:**

Cytokine autoantibodies (c-aAb) have been associated with pulmonary diseases, including severe novel coronavirus disease 2019 (COVID-19) and pulmonary alveolar proteinosis. This study aimed to determine c-aAb association with community-acquired pneumonia (CAP) etiology (SARS-CoV-2, influenza, or bacteria) and c-aAb associations with CAP-related clinical outcomes and pulmonary comorbidities.

**Methods:**

In a cohort of 665 patients hospitalized with CAP, c-aAb targeting interferon α (IFNα), IFNβ, IFNγ, interleukin-1α (IL-1α), IL-6, IL-10, and granulocyte-macrophage colony-stimulating factor (GM-CSF) were measured in plasma samples. Associations between c-aAb and baseline characteristics, pulmonary comorbidities, pathogen, intensive care unit (ICU) transferal, time to clinical stability, and mortality were estimated, with results stratified by sex.

**Results:**

More men infected with SARS-CoV-2 were had high-titer type 1 IFN c-aAb compared to other pathogens. Among patients with CAP, asthma and bronchiectasis comorbidities were associated with high-titer GM-CSF c-aAb in men, and men with high-titer IFNβ c-aAb had increased odds for ICU transferal. High-titer IL-10 c-aAb were associated with faster clinical stability in women

**Conclusion:**

In men with CAP, various c-aAb—including type 1 IFN and GM-CSF c-aAb—were associated with adverse clinical events and comorbidities, whereas c-aAb targeting an autoinflammatory cytokine were associated with a positive outcome in women. This suggests that the potentially immunomodulatory effects of c-aAb depend on pathogen, autoantibody specificity, comorbidity, and sex.

## Introduction

Community-acquired pneumonia (CAP) is a significant global concern and responsible for a considerable amount of morbidity and mortality (1, 2). Pro- and anti-inflammatory cytokines modulate the immune response to CAP, with excessive or impaired cytokine responses associated with increased risk of CAP mortality (3–5).

High titers of neutralizing cytokine autoantibodies (c-aAb) are associated with autoimmunity and immunodeficiency, and increasingly recognized as risk factors for severe viral, bacterial, and mycotic infections in various patient cohorts (6). Furthermore, c-aAb may persist at high-titer levels for several years (7–9). High titers of neutralizing c-aAb targeting type I interferons (IFNs) disrupt antiviral immunity and predict the development of severe coronavirus disease 2019 (COVID-19) caused by the severe acute respiratory syndrome coronavirus 2 (SARS-CoV-2) and influenza virus pneumonia (10–15). Furthermore, c-aAb targeting IFNγ, interleukin (IL)-6, and granulocyte-macrophage colony-stimulating factor (GM-CSF) have been associated with increased susceptibility to various infections in humans such as non-tuberculosis mycobacteria, *Staphylococcus aureus*, and aspergillus (6, 16–18), and GM-CSF c-aAb are counted as a diagnostic factor for pulmonary alveolar proteinosis (PAP) (19). However, the relationship between c-aAb and CAP, including causal pathogens and clinical outcomes, remains undetermined. Additionally, whether c-aAb are associated with other chronic diseases common to CAP patients—such as chronic obstructive pulmonary disease (COPD) or asthma—has not been established (20).

Therefore, this exploratory study compared distributions of c-aAb targeting the cytokines IFNα, IFNβ, IFNγ, IL-1α, IL-6, IL-10, and GM-CSF in patients hospitalized with CAP with registered CAP etiology. We further assessed whether the presence of these c-aAb was associated with specific patterns of pulmonary comorbidities within the cohort (asthma, bronchiectasis, COPD, and fibrosis), as well as whether c-aAb served as predictors of clinical outcomes (including mortality, disease severity, time to readmission, admission to intermediary or intensive care, and time to clinical stability). As both c-aAb distribution and associations to disease outcome have been found to be highly sex-specific (11, 21), we stratified our investigations by sex.

## Methods

### Study reporting

The Strengthening the Reporting of Observational Studies in Epidemiology (STROBE) guidelines for reporting observational cohort studies were used for this study (22).

### Design and settings

This study included patients enrolled between January 2019 and November 2021 in the Surviving Pneumonia Study cohort, an ongoing observational, prospective cohort of patients hospitalized with CAP at Copenhagen University Hospital - North Zealand, Denmark (23, 24). The patients included in this study were followed for at least 1 year after being discharged or until death (**Supplementary Table 1**).

### Participants

Inclusion criteria were age 18 years or older, clinical symptoms or signs consistent with a respiratory infection (e.g., coughing, production of sputum, fever or hypothermia, chest pain, difficulty breathing, and abnormal chest auscultation), and a new infiltrate on a chest x-ray or chest computed tomography scan. Eligible patients were enrolled within 24 h of being admitted to the hospital. The only exclusion criterion was a missing biobank sample, required to measure c-aAb.

### Data collection

Clinical information, including radiology reports, vital signs at admission, comorbidities, microbiological test results, and disease outcomes, was obtained from the electronic medical record. The Charlson comorbidity index, which assigns a weight to 19 specific comorbidities, was used to assess the comorbidity burden (26). These comorbidities included myocardial infarction, congestive heart failure, peripheral vascular disease, cerebrovascular disease, dementia, rheumatic disease, peptic ulcer disease, diabetes without chronic complication, diabetes with chronic complication, hemiplegia or paraplegia, renal disease, any solid malignancy without metastasis, metastatic solid tumor, leukemia or lymphoma, mild liver disease, moderate or severe liver disease, and AIDS/HIV. In addition, data on chronic pulmonary diseases (asthma, bronchiectasis, COPD, and fibrosis) were also collected. Body mass index (BMI) was calculated from self-reported height and weight measured on an electric scale (Seca, Hamburg, Germany) within 48 h of admission. Vital signs included the first measurements of blood pressure, respiratory rate, and body temperature. CAP severity was assessed with the CURB-65 score, which is a risk-stratification tool that assigns one point each for confusion, urea >7 mmol/L, respiratory rate ≥30/min, systolic blood pressure <90 mm Hg or diastolic ≤60 mm Hg, and age ≥65 years. Scores are categorized as 0–1 for mild, 2 for moderate, and 3–5 for severe CAP (27). Plasma samples were prepared from venous blood collected in ethylene diamine tetraacetic acid (EDTA) tubes within 24 h of study enrollment and stored at −80°C until analysis.

### Microbiological testing

Routine microbiological sampling and testing included blood cultures and culturing of respiratory samples (sputum and tracheal aspirate). Polymerase chain reaction testing of respiratory samples (sputum, tracheal aspirate, and oropharyngeal swabs) for causes of atypical pneumonia (*Legionella pneumophila, Mycoplasma pneumoniae*, and *Chlamydia pneumoniae*) and respiratory viruses [influenza A and B viruses, SARS-CoV-2, respiratory syncytial virus (RSV), rhinovirus, enterovirus, human metapneumovirus, parainfluenza virus, and adenoviruses] was also performed. Furthermore, urinary antigen tests for *Streptococcus pneumoniae* (PUT) and *L. pneumophila* (LUT) were performed on some patients. As microbiological testing was conducted at the discretion of the attending physician, the extent of microbiological testing differed between patients.

### Cytokine autoantibody screening

An in-house assay was used to screen plasma samples for c-aAb targeting GM-CSF, IFNα, IFNβ, IFNγ, IL-1α, IL-6, and IL-10 according to a previously described protocol (28). Briefly, plasma samples were diluted 10-fold in a mix of assay buffer and seven different MagPlex microspheres, conjugated to recombinant, human molecules of the c-aAb relevant cytokines. Samples + beads were incubated with 800 rpm shaking for 1 h, followed by rounds of washing with assay buffer using manual magnetic plate inversion. Following a further 30-min incubation with PE-conjugated anti F(ab′)2 IgG, samples were washed again and read on a Luminex 200 system. The assay was performed using room temperature ingredients and shielded from light exposure as much as possible.

Patients were considered to have high titers of c-aAb if their mean fluorescence intensities (MFIs) were above the 90th or 95th percentiles at baseline or the earliest available sample for the seven individual c-aAb. This cutoff was considered to correspond to patients most likely to have functionally cytokine neutralizing c-aAb.

### Outcomes

The outcomes of interest were differences in c-aAb across pathogen groups, baseline characteristics, clinical outcomes, and pre-existing comorbidities in patients. The clinical outcomes were mortality (within 30 or 180 days from admission), transferal to the intermediary or intensive care unit (IMU/ICU), readmission (within 30 or 180 days from discharge), disease severity, and time to clinical stability. Clinical stability was defined according to a modified version of Halm’s criteria (29), which required meeting all of the following parameters concurrently: temperature ≤ 37.2°C, heart rate ≤ 100 beats/min, respiratory rate ≤ 24 breaths/min, systolic blood pressure ≥ 90 mm Hg, and oxygen saturation ≥ 90% without oxygen therapy. Time to clinical stability was measured in hours elapsed since admission.

### Statistical analyses

#### C-aAb vs. baseline characteristics

Continuous variables were summarized as the median with interquartile range (IQR) and categorical variables were summarized as counts with percentages. Differences in c-aAb at admission between sexes in the cohort were compared with Wilcoxon rank sum test for the non-normally distributed c-aAb MFI values and the chi-squared test for comparing the number of cases above/below high-titer cutoffs in the cohort (90th or 95th percentile c-aAb MFI for men or women, respectively). Subsequent analyses were stratified by sex as indicated, as well as performed for men and women combined due to the limited sample size.

Differences in associations between c-aAb MFI and baseline characteristics were estimated with the Mann–Whitney *U* test for dichotomous, categorical variables and by Spearman’s correlation coefficient for continuous variables, respectively, due to the nonparametric distribution of c-aAb. Based on these analyses, variables with significant associations to c-aAb, including age, sex, BMI, all forms of tobacco usage (defined as any prior or current usage of cigarettes, cigarillos, or pipe), and Charlson comorbidity index, were used as covariates in subsequent multivariate analyses.

#### C-aAb vs. pathogens

C-aAb differences between pathogen groups were estimated with the chi-squared test for categorical variables (high titers above the 90th percentile) and Kruskal–Wallis tests followed by Dunn’s *post-hoc* test comparing overall c-aAb distribution between pathogen groups. Pathogen groups were defined as patients with bacterial infections, COVID-19 infection, or other viral infections. These general groupings were chosen in order to maximize *n* for subsequent analyses, especially considering they would also be stratified by sex. Patients with unknown etiology were excluded from these analyses, as well as cases with multiple different pathogen subtypes, such as patients with mixed bacterial and viral infections. Differences in baseline characteristics and vital parameters at admission between pathogen groups were estimated with the chi-squared test for categorical variables and the Kruskal–Wallis test for continuous variables. Subsequently, multivariate logistic regressions were performed with high-titer c-aAb as predictors, and age, BMI, tobacco usage, and Charlson comorbidity index as covariates, as well as sex when testing for the whole cohort.

#### C-aAb vs. clinical outcomes and comorbidities

The Mann–Whitney *U* test was used to estimate differences in general c-aAb MFI across binary clinical outcomes (readmissions within 30 or 180 days from discharge, mortality within 30 days, 180 days, 1 year, 2 years, or all-cause mortality following index admission, and admission to IMU/ICU) and comorbidity status. Chi-squared analyses used to test for high-titer c-aAb association to the aforementioned dependent variables. Analyses concerning clinical outcomes were limited to patients with follow-up spanning a minimum of 30 days, 180 days, etc. Owing to similar results, ultimately data from analyses spanning 180 days of follow-up were chosen to be reported. Multivariate logistic regressions were performed for c-aAb MFI/high-titer c-aAb as predictors for comorbidity status or clinical outcomes, adjusted for age, BMI, tobacco usage, and Charlson comorbidity index. Pathogen grouping was not included as a covariate, as we considered it a possible mediator of potential c-aAb-derived effects. Likewise, analyses were not stratified by pathogen to retain *n*. C-aAb as predictors of disease severity was assessed using an ordinal variable of mild, moderate, or severe CURB-65 score as the dependent variable in logistic regressions, with uni-/multivariate tests utilizing MFI/high-titer c-aAb and the aforementioned covariates as independent variables. As no regressions yielded significant results, we also elected to not include severity as a covariate in other clinical outcome analyses.

The association between c-aAb/high-titer c-aAb and time to death/clinical stability were investigated using the multivariate Cox proportional hazards model, adjusted for covariates as above, with c-aAb and high-titer c-aAb as predictors.

Statistical tests were two-sided, with *p* < 0.05 considered significant, and were conducted using STATA software v.18 (StataCorp, College Station, Texas, USA). Analyses were corrected for multiple testing using Bonferroni correction.

## Results

### Study population and microbiological findings

**Figure 1:**
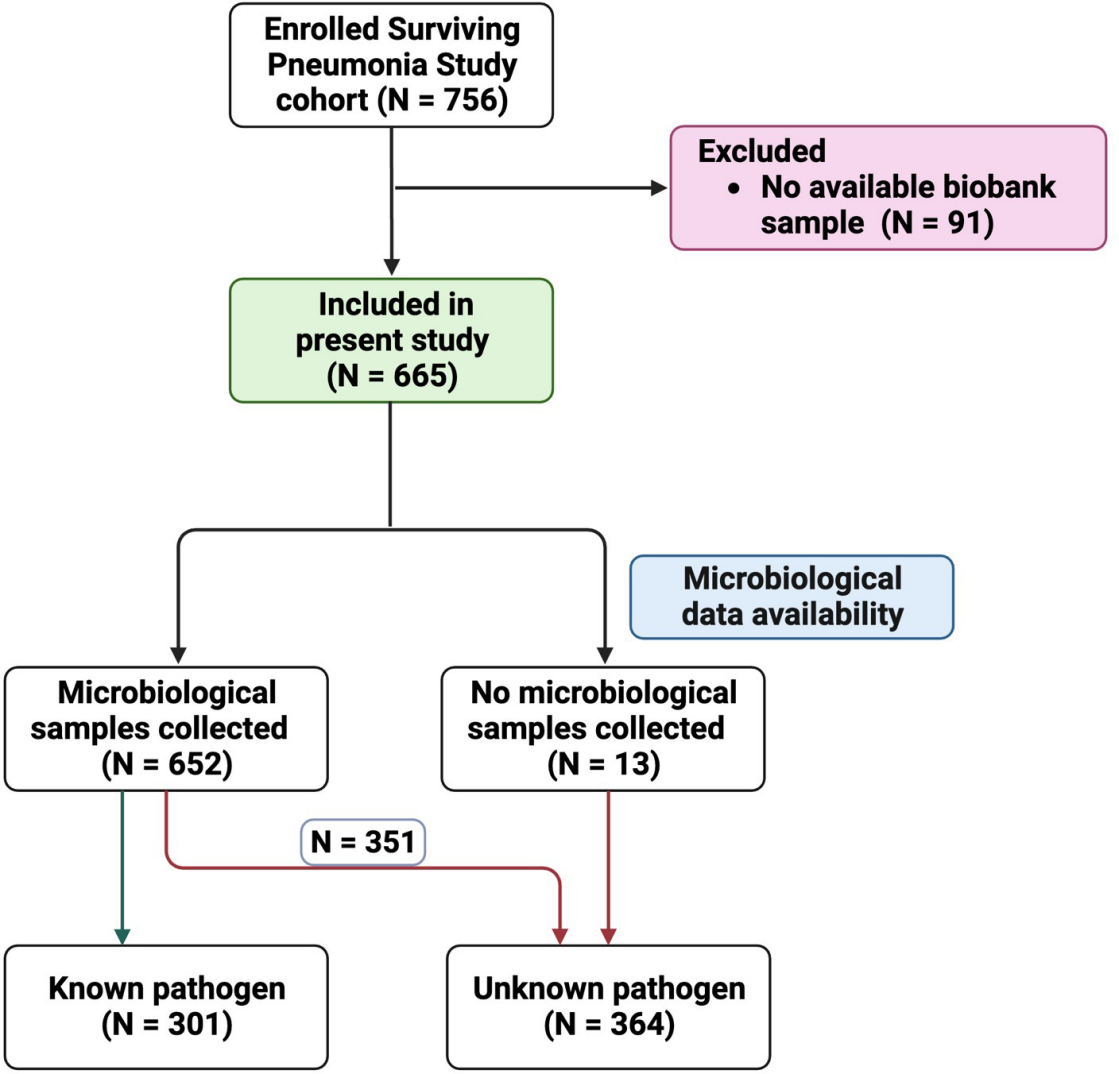
Flowchart of patient inclusion with illustration of patients with known vs. unknown pathogen.

During the study period, 756 patients were enrolled in the Surviving Pneumonia Study cohort, with 91 excluded due to lack of a biobank sample (**Figure 1**). Patient characteristics of the included 665 patients are presented in **Table 1**. Based on the CURB-65 score, 369 (55%) patients presented with mild CAP, while 296 (45%) had moderate to severe CAP (**Table 1**). Of the total number of included patients, 85 (13%) had COVID-19, 39 (6%) had a non-COVID-19 virus, 151 (22%) had a bacterial infection, and 26 (4%) had a bacterial–viral co-infection (**Supplementary Table 2**). Among the 364 (55%) patients with no identified pathogen, 13 had no samples collected for microbiological testing (respiratory samples, blood cultures, or urine samples for LUT and PUT). Further details about this cohort have been published elsewhere (23, 24).

**Table 1:** Baseline characteristics and epidemiologic overview of the Surviving Pneumonia Study cohort (*n* = 665).

Variable	Distribution
Epidemiological variables	
Age, median (IQR), yrs.	73 (63; 81)
Sex, n(%) - Female - Male	314 (47%) 351 (53%)
Tobacco usage, n (%)	442 (66%)
BMI, median (IQR), kg/m^2^	26 (22; 30)
Number of comorbidities	
0, n (%)	41 (6%)
1, n (%)	38 (6%)
≥2, n (%)	563 (88%)
Disease outcome	
IMU admission, n (%)	14 (2%)
ICU admission, n (%)	29 (5%)
Length of stay, median (IQR), days	5 (3;9)
30-day readmission, n (%)	133 (20%)
180-day readmission, n (%)	241 (36%)
30-day mortality, n (%)	62 (9%)
180-day mortality, n (%)	119 (18%)
1-year mortality, n (%)	148 (22%)
2-year mortality, n (%)	184 (28%)
All-course mortality, n (%)	186 (28%)
Death during hospital admission, n (%)	43 (6%)
Time to clinical stability, median (IQR), hrs.	97 (48; 177)
Achieved clinical stability, n of eligible (%)	444/572 (78%)
CURB-65 score	
0, n (%)	135 (20%)
1, n (%)	234 (35%)
2, n (%)	216 (32%)
3, n (%)	68 (10%)
>4, n (%)	12 (2%)
Disease pathogen	
Bacterial infection, n (%)	141 (23%)
COVID-19 infection, n (%)	85 (13%)
Other viral infection, n (%)	39 (6%)
Bacterial/ COVID-19 infection, n (%)	12 (2%)
Bacterial/other viral infection, n (%)	14 (2%)
Unknown, n (%)	364 (52%)

Men exhibited a higher median MFI of IL-1α-, IFNα-, and IFNγ-specific c-aAb and a lower MFI of IL-6 c-aAb compared to women among patients with CAP (**Table 2**). Subsequent c-aAb analyses were stratified by sex.

**Table 2:** Sex differences in c-aAb.

C-aAb specificity	Distribution	Men (n =351)	Women (n = 314)	p-value
IL-1α	C-aAb, median (IQR), net MFI	156 (113; 272)	143 (102; 229)	0.045*
	90^th^ percentile cutoff (n), net MFI	620 (35)	436 (31)	0.068 **
	95^th^ percentile cutoff (n), net MFI	1,389 (18)	795 (16)	0.075***
IFNβ	C-aAb, median (IQR), net MFI	78 (61; 119)	75 (57; 111)	0.116*
	90^th^ percentile cutoff (n), net MFI	224 (35)	274 (31)	0.725 **
	95^th^ percentile cutoff (n), net MFI	497 (18)	405 (16)	0.281 ***
IFNγ	C-aAb, median (IQR), net MFI	356 (245; 719)	306 (213; 472)	<0.001*
	90^th^ percentile cutoff (n), net MFI	1,572 (35)	1,120 (31)	0.232 **
	95^th^ percentile cutoff (n), net MFI	3,287 (18)	3,054 (16)	0.710 ***
IFNα	C-aAb, median (IQR), net MFI	299 (141; 1248)	186 (110; 624)	<0.0001*
	90^th^ percentile cutoff (n), net MFI	6,313 (35)	3,490 (31)	0.015**
	95^th^ percentile cutoff (n), net MFI	9,451 (18)	6,882 (16)	0.153***
IL-6	C-aAb, median (IQR), net MFI	399 (247; 822)	518 (257; 1216)	0.006*
	90^th^ percentile cutoff (n), net MFI	2,463 (35)	3,760 (31)	0.101**
	95^th^ percentile cutoff (n), net MFI	4,895 (18)	6,394 (16)	0.739***
IL-10	C-aAb, median (IQR), net MFI	89 (67; 139)	90 (60; 129)	0.312*
	90^th^ percentile cutoff (n), net MFI	270 (35)	223 (31)	0.180**
	95^th^ percentile cutoff (n), net MFI	521 (18)	471 (16)	0.710***
GM-CSF	C-aAb, median (IQR), net MFI	74 (53; 154)	75 (51; 127)	0.540*
	90^th^ percentile cutoff (n), net MFI	446 (35)	582 (31)	0.260**
	95^th^ percentile cutoff (n), net MFI	2,481 (18)	2,007 (16)	0.469***

*Wilcoxon rank sum test comparing non-normally distributed variables between sexes.

**Chi-squared test comparing testing for c-aAb distribution above/below the full cohort 90th percentile between sexes.

***Chi-squared test comparing testing for c-aAb distribution above/below the full cohort 95th percentile between sexes.

#### C-aAb distribution vs. patient pathogen

**Figure 2:**
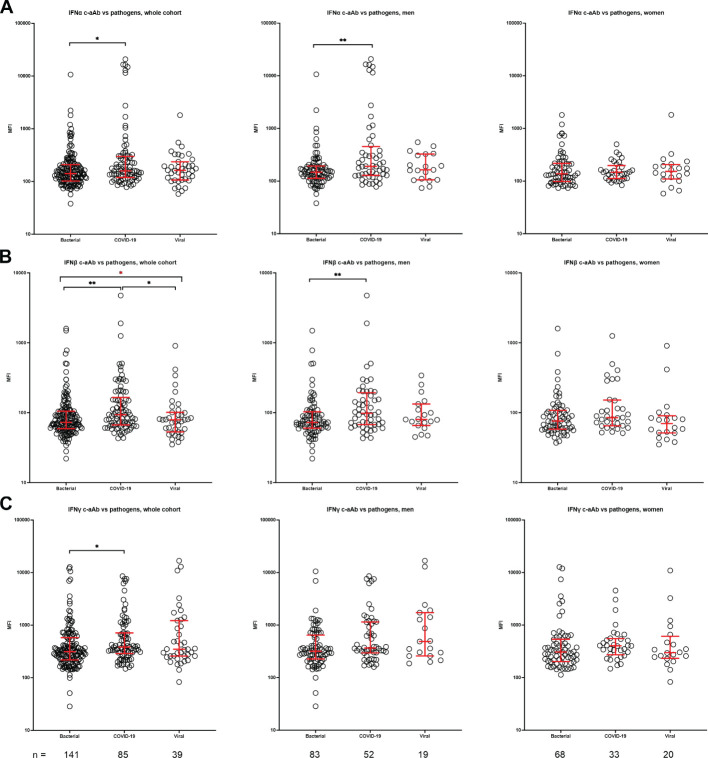
C-aAb in the Surviving Pneumonia Study cohort by pathogen in whole cohort, men and women. Distribution of IFNα **(A)**, IFNβ **(B)** and IFNγ **(C)** specific c-aAb in specific pathogen groups was compared to the rest of cohort using Kruskal–Wallis test, followed by Dunn’s post-hoc test. Red lines indicate median with IQR. * Indicates adjusted *p* < 0.05, **p < 0.01; Red * indicates Kruskal–Wallis test results.

The distribution of c-aAb differed significantly across the pathogen groups for IFNα, IFNβ, and IFNγ c-aAb in the Surviving Pneumonia study cohort (**Figures 2A–C**), with the distribution skewed higher for the COVID-19 group in relation to the bacterial pathogen group. This was also observed for IFNα and IFNβ, specifically for men (**Figures 2A, B**).

Men with COVID-19 or other viral infections expressed more high-titer IFNα c-aAb (**Table 3A**) and IFNγ c-aAb (**Table 3B**), respectively, compared to patients with bacterial infection. In addition, high-titer IFNβ c-aAb presence was borderline significantly elevated in men with COVID-19 following multiple correction (**Table 3C**).

**Table 3A:** MR Analysis of the Mediation Role of Immune Cells in the Relationship between Gut Microbiota and Coagulation Defects, Purpura, and Other Hemorrhagic.

Group and test			Bacterial/Not bacterial	COVID-19/No COVID-19	Virus/no virus (excl. COVID-19)
Whole cohort	C-Ab high-titer Distribution^*^	Pathogen cases	12/151 (8.00%)	14/97 (14.4%)	<5/26 (<19%)
		Non cases	14/124 (11.3%)	17/223 (7.6%)	37/249 (14.9%)
	Test**	Odds Ratio	0.99	1.87	0.33
		*p* value	0.190	0.186	0.295
		Multiple testing adjusted *p* value	0.570	0.558	0.885
Women	C-Ab high-titer Distribution^*^	Pathogen cases	17/68 (10.3%)	<5/39 (<12.5%)	<5/20 (<25%)
		Non cases	<5/53 (<10%)	8/93 (8.6%)	7/101 (6.9%)
	Test**	Odds Ratio	(All cases are high-titer)	0.28	(No cases are high-titer)
		*p* value		*0.264*	
		Multiple testing adjusted *p* value		0.792	
Men	C-Ab high-titer Distribution^*^	Pathogen cases	5/83(6%)	13/58 (22.4%)	<5/19 (<25%)
		Non cases	13/71(18%)	7/111 (6.3%)	17/135 (12.6%)
	Test**	Odds Ratio	0.20	4.30	0.67
		*p* value	0.067	0.016	0.718
		Multiple testing adjusted *p* value	0.201	0.048	1

Binary high-titer/non- high-titer c-aAb (MFI over/under 90th percentile) variables used as predictors for having each pathogen compared to rest of cohort.

* Distribution = n & percentage of high-titer individuals within/outside of pathogen group (case/non case)

** Multivariate regression = logistic regression, with pathogen/non pathogen as outcome, and high-titer c-aAb status as predictor. Age, BMI, tobacco usage, and comorbidity score were used as covariate predictors, as well as sex in the non-stratified analyses. Odds ratio, unadjusted *p* values and multiple testing adjusted *p* values are listed.

**Table 3B:** High-titer IFNβ c-aAb vs. pathogen.

Group and test			Bacterial/Not bacterial	COVID-19/No COVID-19	Virus/no virus (excl. COVID-19)
Whole cohort	C-Ab high-titer Distribution^*^	Pathogen cases	11/151 (7.3%)	16/97 (16.5%)	<5/30 (<17%)
		Non cases	19/124 (15.3%)	19/223 (8.5%)	35/245 (14.3%)
	Test**	Odds Ratio	0.34	2.63	0.67
		*p* value	0.034	0.035	0.614
		Multiple testing adjusted *p* value	0.102	0.115	1
Women	C-Ab high-titer Distribution^*^	Pathogen cases	5/68 (7.4%)	7/39 (18%)	<5/20 (<25%)
		Non cases	9/53 (17%)	8/93 (8.6%)	12/101 (11.9%)
	Test**	Odds Ratio	0.39	1.58	1.32
		*p* value	0.218	0.153	0.753
		Multiple testing adjusted *p* value	0.654	0.459	1
Men	C-Ab high-titer Distribution^*^	Pathogen cases	6/83 (7.2%)	9/58 (15.5%)	<5/19 (<25%)
		Non cases	10/71 (14.1%)	9/111 (8.1%)	14/135 (10.4%)
	Test**	Odds Ratio	0.31	4.63	(No cases are high-titer)
		*p* value	0.086	0.017	
		Multiple testing adjusted *p* value	0.258	0.051	

Binary high-titer/non- high-titer c-aAb (MFI over/under 90th percentile) variables used as predictors for having each pathogen compared to rest of cohort.

* Distribution = n & percentage of high-titer individuals within/outside of pathogen group (case/non case)

** Multivariate regression = logistic regression, with pathogen/non pathogen as outcome, and high-titer c-aAb status as predictor. Age, BMI, tobacco usage, and comorbidity score were used as covariate predictors, as well as sex in the non-stratified analyses. Odds ratio, unadjusted *p* values and multiple testing adjusted *p* values are listed.

**Table 3C:** High-titer IFNγ c-aAb vs. pathogen.

Group and test			Bacterial/Not bacterial	COVID-19/No COVID-19	Virus/no virus (excl. COVID-19)
Whole cohort	C-Ab high-titer Distribution^*^	Pathogen cases	11/151 (7.3%)	13/97 (13.4%)	7/30 (23.3%)
		Non cases	19/124 (15.3%)	19/204 (9.3%)	32/245 (13.1%)
	Test**	Odds Ratio	0.42	1.15	2.89
		*p* value	0.088	0.770	0.048
		Multiple testing adjusted *p* value	0.264	1	0.136
Women	C-Ab high-titer Distribution^*^	Pathogen cases	<5/83 (<6%)	<5/39 (<12.5%)	<5/20 (<25%)
		Non cases	14/71 (19.7%)	10/93 (10.8%)	11/101 (10.9%)
	Test**	Odds Ratio	0.51	1.24	2.04
		*p* value	0.342	0.766	0.354
		Multiple testing adjusted *p* value	1	1	1
Men	C-Ab high-titer Distribution^*^	Pathogen cases	<5/83 (<6%)	9/58 (15.5%)	5/19 (26.3%)
		Non cases	14/71 (19.7%)	9/111 (8.1%)	12/135 (8.9%)
	Test**	Odds Ratio	0.22	0.83	6.45
		*p* value	0.071	0.792	0.005
		Multiple testing adjusted *p* value	0.213	1	0.015

Binary high-titer/non- high-titer c-aAb (MFI over/under 90th percentile) variables used as predictors for having each pathogen compared to rest of cohort.

* Distribution = n & percentage of high-titer individuals within/outside of pathogen group (case/non case)

** Multivariate regression = logistic regression, with pathogen/non pathogen as outcome, and high-titer c-aAb status as predictor. Age, BMI, tobacco usage, and comorbidity score were used as covariate predictors, as well as sex in the non-stratified analyses. Odds ratio, unadjusted *p* values and multiple testing adjusted *p* values are listed.

**Table 4:** GM-CSF c-aAb vs. pulmonary comorbidities.

Group and test				Asthma(n= 87/665)	Bronchiectasis(n= 21/665)	COPD(n= 221/663)	Fibrosis(n= 16/665)
Whole cohort	Continuous c-aAb MFI*	Distribution	Comorbidity cases	75.5 (50, 236)	73 (53, 271)	72 (50, 132)	74.25 (51.75, 157)
			Non cases	73.72 (52, 134)	74.75 (52, 138)	76 (53, 146)	74.25 (52, 138)
		Test	Odds ratio	1.13 (1.02; 1. 23)	0.88 (0.56; 1.39)	1.02(0.91; 1.14)	0.99(0.76; 1.30)
			p value	0.018	0.608	0.748	0.978
			Multiple testing adjusted p value	0.072	1	1	1
	High-titer c-aAb**	Distribution	Comorbidity cases	17/87(19,5%)	<5/21(24%)	22/221(9,9%)	<5/16(<31%)
			Non cases	50/578(8.7%)	63/644(9.8%)	45/442(10.2%)	64/649(9.9%)
		Test	Odds ratio	2.78(1.41; 5.47)	2.49(0.77; 8.04)	1.10(0.54; 2.24)	1.21 (0.25; 5.89)
			p value	0.003	0.128	0.792	0.815
			Multiple testing adjusted p value	0.012	0.384	1	1
Women	Continuous c-aAb MFI*	Distribution	Comorbidity cases	74.5 (51, 118)	53.5 (40, 66)	67 (46, 132)	78.5 (47, 91)
			Non cases	75.5 (50, 236)	76 (51.5, 130)	76.5 (55, 126.5)	75 (51, 130)
		Test	Odds ratio	1.13 (0.99; 1. 29)	0.00003 (2.14e-13; 6447)	1.06(0.87; 1.30)	0.01(3.21e-09; 33992)
			p value	0.149	0.292	0.512	0.551
			Multiple testing adjusted p value	0.447	0.876	1	1
	High-titer c-aAb**	Distribution	Comorbidity cases	9/57(15.8%)	0/10(0%)	13/108(12%)	0/6(0%)
			Non cases	22/257(8.6%)	<5/11(<45,5%)	18/204(8.8%)	31/308(10.1%)
		Test	Odds ratio	2.43(0.96; 6.15)	(No cases are high-titer)	0.71 (0.25; 2.02)	(No cases are high-titer)
			p value	0.060		0.525	
			Multiple testing adjusted p value	0.180		1	
Men	Continuous c-aAb MFI*	Distribution	Comorbidity cases	76 (50, 197)	271 (72, 868)	73 (54, 128)	74.25 (53, 978)
			Non cases	73 (53, 149)	73 (52.5, 147.5)	75 (52, 165.2)	74 (53, 149)
		Test	Odds ratio	1.12 (0.99; 1. 25)	0.99 (0.69; 1.38)	0.99 (0.86; 1.15)	1.03 (0.81; 1.31)
			p value	0.089	0.910	0.998	0.788
			Multiple testing adjusted p value	0.384	1	1	1
	High-titer c-aAb**	Distribution	Comorbidity cases	6/30(20%)	<5/11(<45,5%)	8/113(7.1%)	<5/10(50%)
			Non cases	29/321(9%)	31/340(9.1%)	27/238(11.3%)	32/341(9.4%)
		Test	Odds ratio	3.92 (1.34; 11.26)	8.89 (2.05; 38.49)	1.34(0.49; 3.71)	2.78(0.47; 16.62)
			p value	0.011	0.003	0.564	0.261
			Multiple testing adjusted p value	0.044	0.012	1	0.783

* C-aAb MFI/1000 used as independent variable. Distribution = median + IQR in case/non-case groups, test = logistic regression, with age, BMI, tobacco usage and comorbidity score as covariates, as well as sex in the non-stratified analyses. ** C-aAb high-titer status (>90^th^ percentile) used as independent variable. Distribution = n & percentage of high-titer individuals within/outside of comorbidity group (case/non case), test = logistic regression, with age, BMI, tobacco usage and comorbidity score as covariates, as well as sex in the non-stratified analyses. Outcome was Odds Ratio with 95% Confidence interval. Unadjusted as well as multiple testing adjusted p values are listed.

#### Association of C-aAb with pulmonary comorbidities and clinical outcomes

Regarding the pulmonary comorbidities of CAP patients (asthma, bronchiectasis, COPD, and fibrosis), we observed a higher likelihood for having asthma for high-titer GM-CSF c-aAb, both generally [OR = 2.78 (IQR: 1.41–5.47), *p* = 0.012] and for men specifically [OR = 3.92 (IQR: 1.34–11.26), *p* = 0.044]. Likewise, men with high-titer GM-CSF c-aAb had a significantly higher likelihood for bronchiectasis [OR = 8.89 (IQR: 2.05–38.49), *p* = 0.012, **Table 4**]. High-titer IL-6 c-aAb were associated with increased odds for pulmonary fibrosis diagnosis in men [OR = 8.90 (IQR: 1.65–48.18), *p* = 0.040]. No statistically significant trends were observed between c-aAb and non-pulmonary comorbidities.

**Figure 3:**
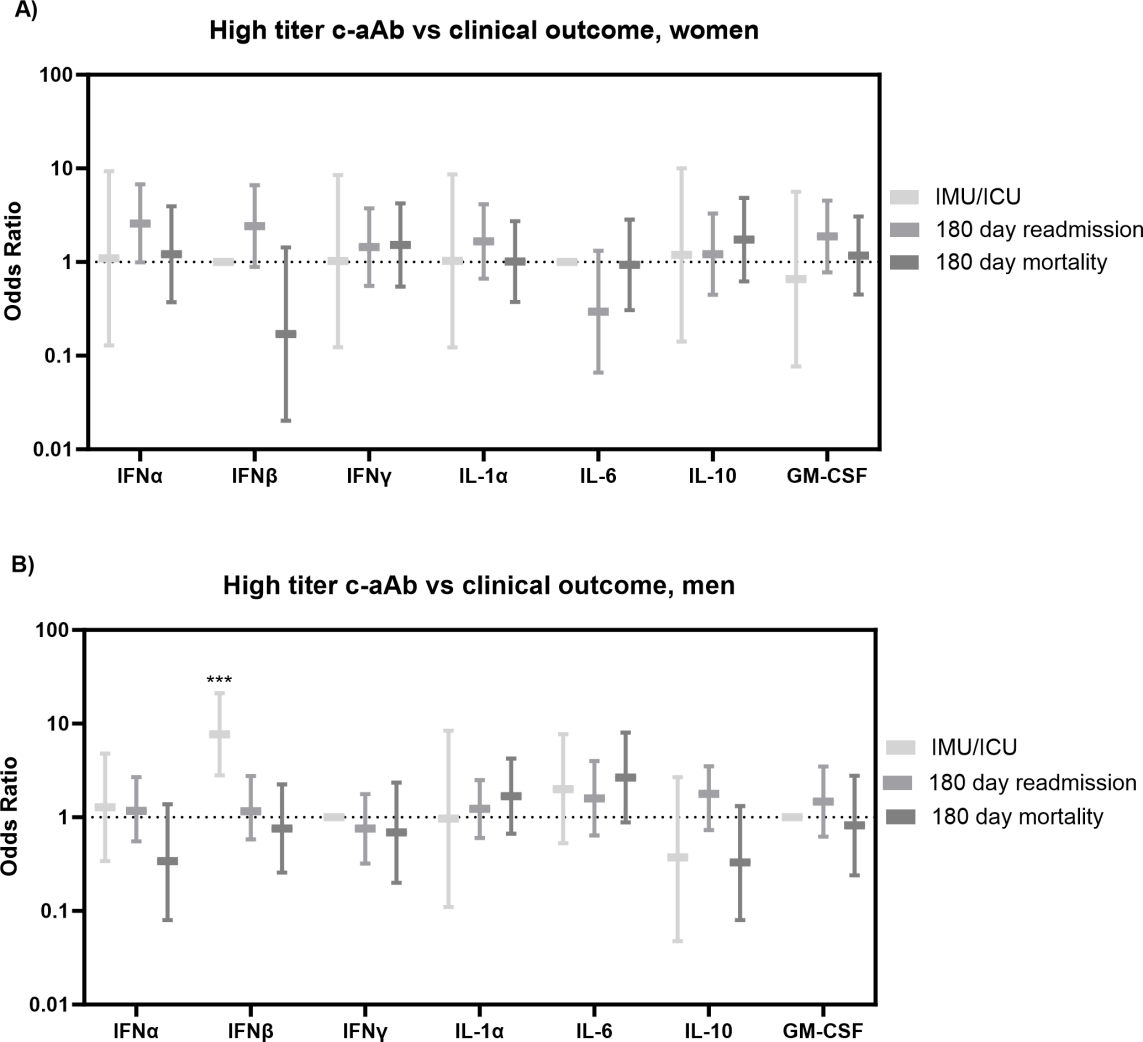
Clinical outcomes vs. high-titer c-aAb in the Surviving Pneumonia Study cohort. Logistic regression with IMU/ICU transferal, 180 days readmission, or mortality (180 days) as outcomes for women **(A)** and men **(B)**. Covariates were age, BMI, sex, tobacco, Charlson comorbidity index. *** indicates *p* <0.001.

In terms of clinical outcomes (mortality, readmission, IMU/ICU admission, and time to clinical stability), high-titer IFNβ c-aAb in men predicted increased odds of admission to the IMU or ICU [OR = 7.69 (95% CI: 2.80–21.12), *p* < 0.001, **Figure 3**]. When subtracting one major pathogen group (including “Unknown”) at a time from subsequent logistic analyses results, this finding for IFNβ c-aAb was largely unchanged upon exclusion of bacteria, virus, or COVID-19 infection as the cause, but became nonsignificant upon removal of the “Unknown” group (data not shown).

No other c-aAb presented significant associations to outcomes, including for severity, all-cause mortality, and readmission at any of the time points between 30 days and 2 years from baseline (hospital admission for mortality and discharge for readmission).

**Figure 4:**
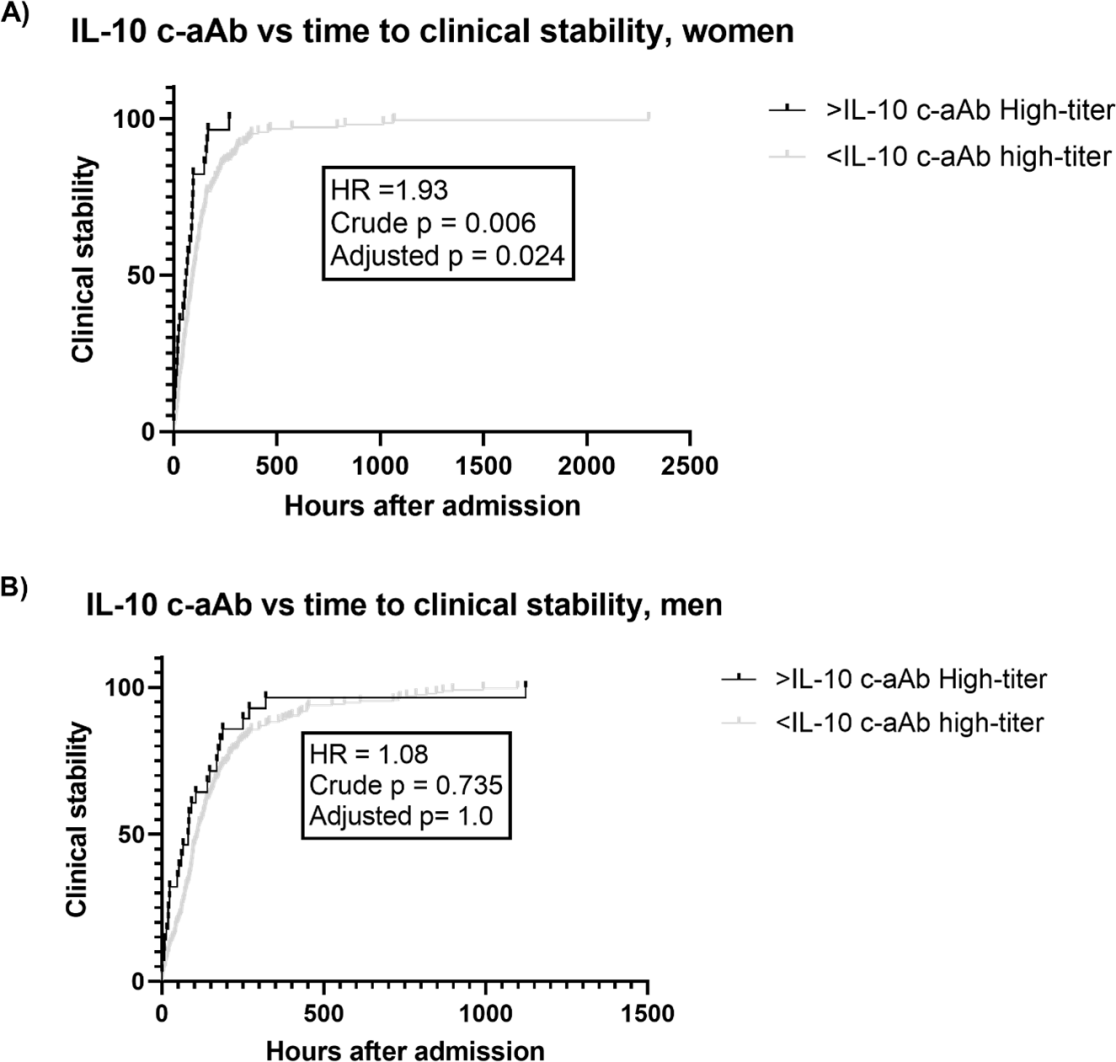
IL-10 c-aAb vs. time to clinical stability in the Surviving Pneumonia Study cohort. Survival curves for time to clinical stability (hours after admission) for individuals with or without high-titer IL-10 c-aAb (90th percentile) for women **(A)** or men **(B)** with time to stability data (572/665). Tested for difference using Cox regression, adjusted for age, BMI, tobacco usage, and Charlson comorbidity index, hazard ratio (HR), crude *p*-value, and multiple testing adjusted *p*-value (×4 for four different clinical outcomes investigated) listed.

Finally, high-titer IL-10 c-aAb were associated with reduced time to clinical stability in women [HR = 1.93 (1.21; 3.08), *p* = 0.024, **Figure 4**].

## Discussion

In this study, we screened for c-aAb targeting IFNα, IFNβ, IFNγ, IL-1α, IL-6, IL-10, and GM-CSF in the Surviving Pneumonia Study cohort, comprising 665 patients with CAP caused by COVID-19, influenza virus, bacteria, or unknown pathogen.

Beyond various autoimmune diseases (6), the currently most well-documented instances of naturally occurring c-aAb as predictors for pathologies are those between GM-CSF c-aAb and PAP and IFNα c-aAb and COVID-19 (11, 19). Numerous observational studies have demonstrated a positive association between the presence of high titers of c-aAb that target type I IFNs, particularly IFNα, and severe disease in patients with COVID-19 or influenza virus pneumonia, with men in particular being at risk (10–13, 30, 31). In PAP, GM-CSF c-aAb-associated macrophage dysfunction can lead to the accumulation of mucus in the airways, and in COVID-19, IFNα c-aAb attenuates the type 1 IFN-mediated antiviral response. Both c-aAb have been detected in bronchiolar lavage (14, 32). This led us to further investigate c-aAb in the context of acute pulmonary disease in our Surviving Pneumonia Study cohort and, furthermore, to look for associations between levels of c-aAb and pulmonary disease-associated comorbidities in CAP patients.

In line with the existing literature, our study found a positive association between IFNα c-aAb titers and patients with COVID-19 infection in comparison to other pathogens, as well as a smaller, less significant association for IFNβ. High-titer IFNγ c-aAb were also more prominent specifically in men with non-COVID-19 viral infection, showcasing that different c-aAb are linked to different pathologies.

The higher MFI levels of c-aAb targeting IL-1α in men compared to women in our study, as well as their association with age, also align with previous studies of Danish blood donors (21). This includes positive associations of IFNα c-aAb and male sex—also documented in COVID-19 studies—as well as a positive association of IL-6 c-aAb and female sex (21, 33, 34).

It is our general hypothesis that high-titer c-aAb may inhibit the functionality of target cytokines and thus modulate any cytokine-associated disease risk and outcomes. Within this study, we found c-aAb targeting IFNβ in men exclusively to be associated with adverse outcomes, namely, increased odds of being admitted to the IMU/ICU. It is unclear what renders IFNβ c-aAb specifically relevant in this context, but IFNβ c-aAb have been found to be associated with critical disease course independently of IFNα c-aAb previously, such as for infection with *S. pneumoniae* (11, 35). In our study, this association to clinical outcomes was not reliant on the COVID-19-infected subset of the cohort, indicating that type 1 IFN targeting c-aAb may be relevant in contexts beyond COVID-19.

In contrast to these findings, we found IL-10 c-aAb to be associated with reduced time to clinical stability for women, the lone case of a c-aAb being associated with a preferable outcome. This is in line with prior findings of IL-10 c-aAb being associated with reduced infection risk in Danish blood donors (36), which we speculate is due to the anti-inflammatory role of IL-10, where c-aAb-mediated inhibition may result in a more active immune system. Conversely, we have also found IL-10 c-aAb to be associated with increased risk of cardiovascular disease in a cohort of kidney transplant recipients, again emphasizing the highly context-specific impact of c-aAb (37).

While GM-CSF c-aAb levels were not associated with clinical outcomes, we observed that men had significant associations of high-titer GM-CSF c-aAb to diagnosed asthma and bronchiectasis. We believe that we are the first to describe these associations.

It has been suggested that tissues expressing high cytokine levels may yield potentially immunogenic alternative cytokine-analogs, due to some post-transcriptional and translationally modified cytokine-analogs escaping negative selection in the thymus (38). Such scenarios might include pulmonary diseases, as GM-CSF is, for instance, highly expressed in bronchoalveolar lavage and sputum of patients with COPD compared to healthy controls (39, 40). It is possible that our association of high-titer GM-CSF c-aAb to asthma and bronchiectasis in our cohort might reflect such a breach of tolerance. Future studies may investigate whether GM-CSF c-aAb influence disease severity for these specific comorbidities, as seen in a murine study where neutralizing GM-CSF-specific antibodies ameliorated inflammation caused by exposure to cigarette smoke, the primary risk factor for COPD (41). Conversely, both asthma and bronchiectasis have been known to feature accumulation of mucus, a key feature of PAP, which could thus also be partially GM-CSF c-aAb derived in these diseases (42, 43). A case with both bronchiectasis and GM-CSF c-aAb-derived PAP has been described (44), yet none of the patients in the cohort featured diagnosed PAP. Thus, based on our findings, we speculate that pulmonary diseases linked causally to high-titer c-aAb—including PAP or similar pathologies—may be overlooked and labeled with other diagnoses, and our study suggests the merit of c-aAb in more full-fledged cohorts for the comorbidities in question. Finally, autoantibody Ig subclass has recently been found correlated to COVID-19 severity (45), and future studies could thus assess the potential links of c-aAb subclasses to clinical outcomes.

To our knowledge, this is the first study to investigate this wide range of c-aAb in the context of CAP and its wider associated pulmonary comorbidities. That our findings are generally in line with previously observed age/sex patterns of c-aAb distribution and disease associations, and seem coherent with the functionality of the targeted cytokines all corroborate our results. Weaknesses of the study include the comparatively small sample sizes of the patients with specific diagnosed comorbidities and the lack of direct neutralization assays for the seven c-aAb screened in our cohort. Another limitation is that the patients being attended to by different physicians may have caused variation in the level of microbiological testing performed, as this was decided at the discretion of the attending physician.

In conclusion, our study underlines the relevance of type 1 IFN targeting c-aAb for COVID-19 patients and shows IFNβ c-aAb in men being specifically associated with unfavorable infectious disease outcomes outside of a COVID-19 context. Conversely, c-aAb targeting the anti-inflammatory cytokine IL-10 were associated with faster clinical stability in women, and GM-CSF-specific c-aAb were found to be associated with asthma and bronchiectasis in men. Taken together, these findings, including their high degree of sex dependency, emphasize that the influence of c-aAb may be highly tied to specific contexts and cytokine functionality, and suggest multiple avenues for future c-aAb studies, including association to multiple pulmonary clinical phenotypes.

## Data Availability

The datasets presented in this article are not readily available because the datasets used for the current study are not publicly available. However, pseudonymised data can be provided by the corresponding author upon a reasonable request. Requests to access the datasets should be directed to jakob.hjorth.von.stemann@regionh.dk.
