# Supplementary material for: Autoantibodies targeting interferons and GM-CSF are associated with adverse outcome risk, comorbidities, and pathogen in community-acquired pneumonia
Source: Front Immunol. 2024 Nov 13;15:1459616. doi: 10.3389/fimmu.2024.1459616 (PMC11598332; doi:10.3389/fimmu.2024.1459616)
Supplement: Supplementary file 1 [file Table1.docx]

**Supplementary tables**

| **Supplementary Table 1: Follow-up in the Surviving Pneumonia cohort** | | | |
| --- | --- | --- | --- |
| **Characteristic** | **Overall, n = 665** | **Alive, n = 479^1^** | **Dead, n = 186^1^** |
| Follow-up 0-1 year | 148 (22%) | 0 (0%) | 148 (80%) |
| Follow-up 1-2 years | 154 (23%) | 118 (25%) | 36 (19%) |
| Follow-up ≥ 2 years | 362 (55%) | 360 (75%) | <5 (<4%) |
| ^1^Indicates the status (alive or dead) of patients 1 year after the last patient was enrolled | | | |

| **Supplementary Table 2: Clinically significant microbiological findings** | |
| --- | --- |
| **Pathogen** | n |
| **Monobacterial infection** | 136 |
| Other gram-negative bacterium (OGNB)* | 45 |
| *Haemophilus influenzae* | 29 |
| *Streptococcus pneumoniae* | 20 |
| *Staphylococcus aureus* | 13 |
| *Legionella pneumophila* | 10 |
| *Mycoplasma pneumoniae* | 7 |
| *Pseudomonas aeruginosa* | 5 |
| *Streptococcus spp.* | <5 |
| *Moraxella catarrhalis* | <5 |
| **Mixed bacterial infection** | 15 |
| *P. aeruginosa*, OGNB | <5 |
| *S. pneumoniae, H. influenzae* | <5 |
| *H. influenzae, L. pneumophila* | <5 |
| *H. influenzae, M. catarrhalis* | <5 |
| *H. influenzae,* OGNB | <5 |
| *M. catarrhalis*, OGNB | <5 |
| *H. influenzae, S. aureus* | <5 |
| *L. pneumophila, S. aureus,* OGNB | <5 |
| *S. aureus, P. aeruginosa, M. catarrhalis*, OGNB | <5 |
| *S. pneumoniae, S. aureus* | <5 |
| *H. influenzae, Streptococcus* spp. | <5 |
| *L. pneumophila, Streptococcus* spp. | <5 |
| *S. aureus, Streptococcus* spp. | <5 |
| **Bacterial and viral co-infection** | 26 |
| *S. pneumoniae,* rhinovirus | <5 |
| *S. pneumoniae,* parainfluenza virus | <5 |
| *H. influenzae,* human metapneumovirus | <5 |
| *H. influenzae,* influenza A virus | <5 |
| OGNB. influenza virus | <5 |
| *P. aeruginosa,* influenza virus | <5 |
| *P. aeruginosa,* respiratory syncytial virus | <5 |
| *S. aureus,* influenza A virus | <5 |
| *S. aureus,* influenza A virus, candidemia | <5 |
| *S. pneumoniae*, human metapneumovirus | <5 |
| *Streptococcus* spp., respiratory syncytial virus | <5 |
| OGNB, SARS-CoV-2, | <5 |
| *S. aureus,* SARS-CoV-2, | <5 |
| Candidemia, SARS-CoV-2, | <5 |
| *P. aeruginosa*, SARS-CoV-2, | <5 |
| *Streptococcus* spp., SARS-CoV-2, | <5 |
| **Viral infection** | 124 |
| SARS-CoV-2 | 85 |
| Influenza A virus | 25 |
| Respiratory syncytial virus | 5 |
| Rhinovirus | <5 |
| Human metapneumovirus | <5 |
| Parainfluenza virus | <5 |
| Adenovirus | <5 |

* Other gram-negative bacteria (OGNB) encompass isolation of *Escherichia coli, Klebsiella pneumoniae, K. oxytoca, Enterobacter* spp.*, Stenotrophomonas maltophilia, Eikenella corrodens, Neisseria meningitidis, Proteus* spp. *or P. mirabilis.*
